# Tumor suppressor micro RNA miR-145 and onco micro RNAs miR-21 and miR-222 expressions are differentially modulated by Hepatitis B virus X protein in malignant hepatocytes

**DOI:** 10.1186/1471-2407-14-721

**Published:** 2014-09-26

**Authors:** Manikankana Bandopadhyay, Arup Banerjee, Neelakshi Sarkar, Rajesh Panigrahi, Sibnarayan Datta, Ananya Pal, Shivram Prasad Singh, Avik Biswas, Shekhar Chakrabarti, Runu Chakravarty

**Affiliations:** ICMR Virus Unit, Kolkata, GB-4, 1st floor, ID & BG Hospital Campus, 57, Dr. S C Banerjee Road, Beliaghata, Kolkata, West Bengal 700010 India; Biodefence & Biodiversity Group, Defence Research Laboratory (DRDO), Tezpur, Assam India; Department of Gastroenterology, SCB medical college, Cuttack, India; National Institute of Cholera and Enteric Diseases, Kolkata, India; Department of Pathology & Lab Medicine, Tulane University School of Medicine, New Orleans, Louisiana 70112 USA

**Keywords:** HBx, Hepatocellular carcinoma, HepG2, HepG2.2.15, microRNA

## Abstract

**Background:**

Hepatitis B Virus (HBV) X protein (HBx) is known to be involved in the initiation and progression of hepatocellular carcinoma (HCC) through modulation of host gene response. Alterations in miRNA expressions are frequently noted in HCC. This study is aimed to examine the role of HBx protein in the modulation of oncogenic miRNA-21, miRNA-222 and tumor suppressor miRNA-145 in malignant hepatocytes.

**Methods:**

Expressions of miRNA-21, miRNA-222 and miRNA-145 were measured in HepG2 cells transfected with HBx-plasmid (genotype D) and with full length HBV genome (genotype D) and also in stably HBV producing HepG2.2.15 cells using real time PCR. Their target mRNAs and proteins - PTEN, p27 and MAP3K - were analyzed by real time PCR and western blot respectively. miRNA expressions were measured after HBx/D mRNA specific siRNA treatment. The expressions of these miRNAs were analyzed in liver cirrhosis and HCC patients also.

**Results:**

The study revealed a down-regulation of miRNA-21 and miRNA-222 expressions in HBx transfected HepG2 cells, pUC-HBV 1.3 plasmid transfected HepG2 cells as well as in HepG2.2.15 cells. Down regulation of miRNA-21 and miRNA-222 expression was observed in patient serum samples. Down regulation of miRNA-145 expression was observed in HepG2 cells transiently transfected with HBx and pUC-HBV1.3 plasmid as well as in patient samples but the expression of miRNA-145 was increased in HepG2.2.15 cells. Target mRNA and protein expressions were modulated in HepG2 cells and in HepG2.2.15 cell line consistent with the modulation of miRNA expressions.

**Conclusion:**

Thus, HBx protein differentially modulated the expression of miRNAs. The study throws light into possible way by which HBx protein acts through microRNA and thereby regulates host functioning. It might suggest new therapeutic strategies against hepatic cancer.

## Background

Hepatocelllular carcinoma (HCC) is one of the most common forms of cancer in the world and chronic Hepatitis B Virus (HBV) infection may result in severe complications as liver cirrhosis (LC) and HCC. HBV X protein (HBx) has been the focus of much attention in recent years because it is thought to play key roles in the development of HCC. It is a multifunctional oncoprotein that alters host gene expression by activating a plethora of cytoplasmic signal transduction pathways (e.g., NF-κB, Src, Ras, AP-1, AP-2, PI3K/Akt, Jak/STAT, Smad and Wnt) [1, 2]. HBx exerts pleiotropic effect as transcriptional transactivator by interacting with nuclear transcription factors (e.g., CREB, ATF-2, Oct-1, TBP) and basal transcription factors [3] contributing to increased cell proliferation and survival [4]. HBx modulates other cellular processes like reduction of DNA repair, impediment of p53-mediated apoptosis by direct interaction with p53 [5], activation of mitogen activated protein kinase (MAPK) pathways and induction of apoptosis by altering the TNFα and NF-κB signaling pathways [6–9].Current information suggests that HBx protein may increase the expression of TERT and telomerase activity, increasing the lifespan of hepatocytes thus transforming to malignancies [10]. Taken together, HBx induces persistent changes in different cellular genes that subsequently provide signal to hepatocytes for growth and proliferation thus leading to the development of HCC.

MicroRNAs (miRNAs) are a newly identified class of functional transcripts in eukaryotic cells [11], which are 21 to 23-nucleotide highly conserved RNA molecules that regulate the stability or translational efficiency of target mRNAs [12]. The pattern of miRNA expression can be correlated with cancer type, stage and other clinical variables. miRNA expression analyses have suggested both oncogenic and tumor-suppressive roles of miRNAs. Widespread differential expression of miRNA genes in malignant tissues compared to normal tissues are well documented [13]. miR-21 [14–17], let-7a [16] and miR-224 [17] are up regulated in HCC. miR-145 is found to be modulated in HuH 7 hepatic cancer cell lines, human HCCs [18] as well as carcinomas from other tissues. Overexpression of miR-221 and miR-222 directly results in down regulation of the tumor suppressor and cell cycle regulator p27 (Kip1) [19].

Recent evidences are emerging about the interaction between HBx protein and miRNAs. In HepG2 cells, HBx induced widespread modulation of miRNAs. Along with the HBx protein, the HBx mRNA acts synergistically to repress miR-15a/16 expression through induction of c-Myc gene [20, 21]. miR-29a was found to be up-regulated by HBx protein which in turn enhances cell migration by targeting PTEN in hepatoma cell lines [22]. miR-101 is down-regulated by the HBx protein and induces aberrant DNA methylation by targeting DNA methyl transferase 3A [23].

As HBx protein is crucially associated with development of HCC and cellular miRNA expressions are shown to be perturbed by viral X protein, we aimed to obtain an insight into the possible role of HBx protein of HBV in the modulation of expressions of two oncomiRNA - miR-21 and miR-222 and one tumor suppressor miRNA - miR-145 in malignant hepatocytes. We observed that expressions of all the candidate miRNA were down- regulated in HepG2 cell line ectopically expressing HBx through transient transfection. This result was validated by transfecting HepG2 cells with 1.3 fold HBV genome. We found differential expression of these miRNAs in stable HBV producing cell line HepG2.2.15. We also demonstrated that target mRNAs of these miRNAs as well as corresponding proteins (PTEN, p27 (Kip1) and MAP3K; targets of miRNA-21, miRNA- 222 and miRNA−145 respectively) were modulated accordingly by quantitative Real Time Polymerase Chain Reaction (qRT-PCR) and western blot respectively. This result encouraged us to undertake further investigation utilizing patient samples. Interestingly, we found reduced expression of these miRNAs in samples from advanced liver disease (LC and HCC) patients.

## Methods

### Cell culture

The hepatoblastoma cell line HepG2 was maintained in Dulbecco’s modified Eagle medium (DMEM) with 10% fetal bovine serum (Sigma Aldrich, Munich, Germany) at 37°C in a humidified atmosphere with 5% CO_2_. After approximately 80% cell confluency was reached, cells were harvested for RNA isolation. HepG2.2.15 cells which are a kind gift of Dr. Tatsuo Kanda, Japan, were maintained in the RPMI1640 medium with 12% fetal bovine serum in a 37°C humid incubator in an atmosphere of 5% CO_2_. The cells were generated every three days and could be used when HBV DNA was detected stably in the supernatant.

### Plasmids and RNA oligonucleotides

HBx plasmid -pCXN2-HBx of genotype D was gifted by Dr. Tatsuo Kanda, Japan. 1.3 fold full length HBV genome (genotype D) cloned into pUC 19 vector was a generous gift of Dr. Mashashi Mizokami, Japan. RNA was extracted 48 hours post transfection. HBx-siRNA [24] was used to produce small interfering RNAs (siRNAs) targeting HBx mRNA (genotype D) (Ambion, TX, USA). siRNA duplexes with non-specific sequences were taken as negative control (NC) for siRNA experiments. siRNA transfection were carried out using Lipofecatmine 2000 (Invitrogen, CA, USA) reagent and medium was replaced 6 hour after transfection. RNA was extracted at 24, 48 and 72 hours post siRNA treatment.

### Study subjects

A total of 89 advanced liver disease subjects were recruited in this study which includes two groups: those with LC and those who had developed HCC. These patients were screened for the presence of HBV DNA and 49 were found to be HBV DNA positive. Among them 37 advanced liver disease patients (LC and HCC) were infected with HBV genotype D. Finally 16 patients of age group of 35–48 years were selected. In addition, 16 age and sex matched healthy individuals were recruited as normal controls. The expression of miRNA-21, miRNA- 222 and miRNA−145 were first compared between 16 advanced liver disease patients and 16 healthy individuals (control). Subsequently advanced liver disease patients were subdivided to LC and HCC patients to indicate the significance of miRNA expression variation in these 2 distinct clinical groups.

The patients were admitted to the SCB Medical College of Orissa, India from April 2012 to December 2012. The signed informed consent was obtained from all the study subjects and the study protocol was approved by Kalinga foundation ethical committee. Patient samples were assigned on arbitrary identification based on the order of enrollment in our study. Study subjects were free of other viral infections, including Human Immunodeficiency virus (HIV), Hepatitis C virus (HCV). Control samples were obtained from voluntary blood donors negative for HIV, HBV and HCV.

### HBV genotype determination

For genotype identification surface gene (partial) of HBV was amplified using an established nested-PCR assay we had previously reported [25]. The amplified products were directly sequenced and phylogenetic analysis was performed for HBV genotype determination.

### Cell transfection

Transfection was performed using Lipofecatmine 2000 (Invitrogen) following manufacturer’s instructions. Briefly, twenty four hours prior to transfection 5 × 10^5^ HepG2 cells were seeded into a six well plate. Cells were transfected with two doses - 1 μg and 2 μg of pCXN2-HBx plasmid, 1.3 fold HBV plasmid (puC19-1.3 HBV) and empty vector. In case of HBx and HBV plasmid transfection, after 48 hours, cells were used for RNA extraction. For siRNA experiments RNA were extracted 24, 48 and 72 hours post transfection.

### RNA isolation from cells and patient samples

Total RNA was extracted using TRIzol reagent (Invitrogen) from 1 × 10^6^ ~ 2 × 10^6^ cells according to manufacturer’s protocol. In case of patient samples total small RNAs were extracted from 400 μl of serum using the mirVanaTM miRNA isolation kit following the manufacturer’s protocol (Ambion). Extracted RNA were eluted with 100 μl of nuclease-free water.

### cDNA synthesis and quantitative mRNA expression by real-time PCR

Reverse transcription was performed using the RevertAid first-strand cDNA synthesis kit following the manufacturer’s instructions (MBI Fermentas, Vilnius, Lithuania). RNA quantity and quality were assessed by determination of the optical density at 260 and 280 nm using spectrophotometry and additional visualization by agarose gel electrophoresis. Real-time PCR was performed in the ABI 7000 SDS (Applied Biosystems, Foster City, CA, USA) using the Power SYBR Green (Applied Biosystems) according to the manufacturer’s instructions. Briefly, cDNA was diluted 5 times and 4 μl diluted cDNA template was used for each PCR with 250 nM forward and reverse primers in a total volume of 20 μl. The thermal cycling conditions comprised 5 min at 95°C, followed by 40 cycles at 95°C for 15 s, 60°C for 30 s. All of the reactions were performed in triplicate. The relative quantity of the target mRNA was normalized to the level of the internal control GAPDH mRNA level. The relative quantitative analyses of the data were performed using 7000 system SDS software v1.2.3 (Applied Biosystems). The relative quantitation of target gene expression was obtained using the comparative ΔΔC_T_ method.

### Western blot analysis

After 48 hours of transfection, proteins were prepared for western blot analysis. Cells were washed in cold PBS and cellular proteins were extracted using NP-40 buffer for 30 minutes at 4°C. Lysates were cleared by centrifugation and proteins were separated by gel electrophoresis. Membranes were blocked in TBS-0.1% Tween 20 (TBS-T)/5% (w/v) milk for 1 hour at room temperature. Membranes were then incubated with primary antibodies diluted in TBS-T for 4 hour at room temperature. Subsequently, membranes were washed with TBS-T and incubated with peroxidase-conjugated secondary antibody diluted in TBS-T at room temperature for 30 minutes. Membranes were washed in TBS-T and bound antibodies were detected by enhanced chemiluminescence system Western Blotting Detection Reagents (Amersham Biosciences, Buckinghamshire, UK). The primary antibodies used were anti-PTEN, anti-p27, anti-MAP3K and anti-β-actin (Santa Cruz, USA). Proteins bands were quantified using Dentiometric scanner (Bio-Rad-GS-800, USA).

### miRNA assay

Approximately, 35 ng of total RNA was reverse-transcribed into a 10-uL volume with the TaqMan miRNA reverse-transcriptase kit (Applied Biosystems) according to the manufacturer’s recommendations. Then, 3 uL of the reverse-transcription reaction was used in each of the real-time PCR assays. Analyses of a subset of miRNAs (miR-21, miR-222 and miR-145) were carried out in triplicates by means of the TaqMan human miRNA assays (Applied Biosystems) using 7000 system SDS software v1.2.3 (Applied Biosystems).

### Statistical analysis

All statistical analyses were performed by using GraphPad Prism (GraphPad Software v5.0, USA). Data from experiments are expressed as the mean ± SD. A test (unpaired, two-tailed) was used for comparison between distributions of genotypes. Nonparametric statistical analysis was performed using the Mann–Whitney U test for unpaired observations. A probability level of p <0.05 was set for statistical significance.

## Results

### Differential expression of miRNA-21, miRNA-222 and miRNA-145 in transiently transfected HepG2 cells with HBx, 1.3 fold HBV genome and in stable HBV producing cell line

**Figure 1:**
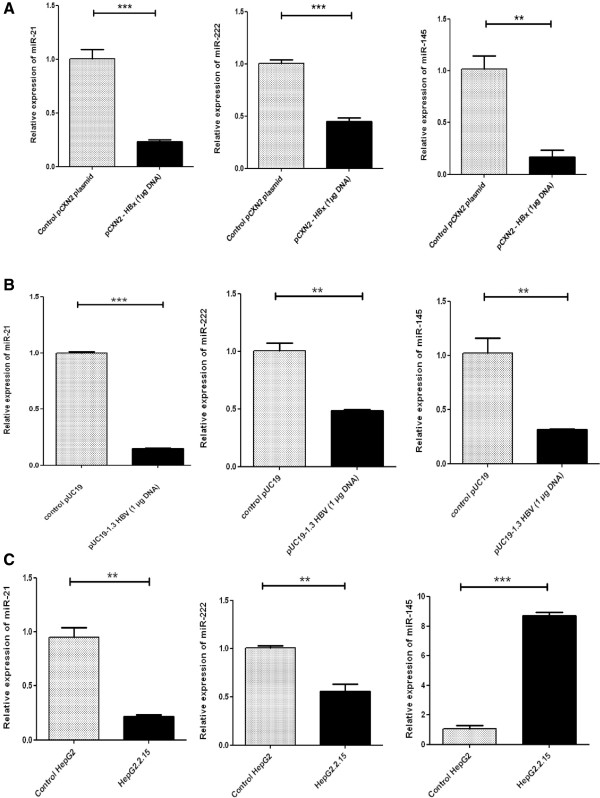
**HBx modulates the expressions of miR-21, miR-222 and miR-145 in hepatoblastoma cells** ***in vitro.*** **(A)** The relative expressions of miR-21, miR-222 and miR-145 in HepG2 cells transiently transfected with HBx expressing plasmid pCXN2-HBx or control vector. Cells were transfected with 1 μg of pCXN2-HBx or pCXN2 as a control. **(B)** The relative expressions of miR-21, miR-222 and miR-145 in HepG2 cells transiently transfected with 1.3 fold full length HBV genome cloned into pUC19 plasmid or control vector. The cells were transfected with 1 μg pUC19-HBV or 1 μg pUC19. **(C)** The relative expressions of miR-21, miR-222 and miR-145 in stably HBV producing HepG2.2.15 cell line or control HepG2 cells. Cells were collected for analysis 48 hour after each transfection. The miRNA expressions were measured by qRT-PCR. Plotted are the mean ± SD of three samples normalized to U6 expression (*P < 0.05, **P < 0.01, ***P < 0.001; Student’s t-test).

All the miRNA on which this study was focused i.e. miRNA-21, miRNA-222 and miRNA-145 were found to be down regulated in HBx transfected HepG2 cells compared to the HepG2 cells transfected with empty expression vector (Figure 1A). The fold change during down regulation of miRNA- 21, miRNA- 222 and miRNA-145 in HBx transfected HepG2 cells compared to HepG2 cells transfected with empty plasmid are presented in Table 1. Transfection of HepG2 cells with 1.3 fold HBV genome supported the findings of our previous experiments. Here also, we found that all the candidate miRNA expressions were reduced in 1.3 HBV transfected cells compared to HepG2 cells transfected with empty pUC19 vector (Figure 1B). When we measured the expression of these three miRNAs in HepG2.2.15 cells which is a stable HBV producing cell line, we found that miR-21 and miR-222 levels are significantly decreased in HepG2.2.15 cell line whereas level of miR-145 was found elevated compared to the control HepG2 cells (Figure 1C). The miRNA expression was normalized using snRNA U6 as internal control. miRNA data were analyzed by the comparative ΔΔC_T_ method.

**Table 1:** **The fold changes (log**_**2**_ **values) during down regulation of miRNA- 21, miRNA- 222 and miRNA-145 in HBx transfected HepG2 cells compared to HepG2 cells transfected with empty expression vector**

miRNA	HepG2 transfected with HBx plasmid (1 μg DNA)	HepG2 transfected with HBx plasmid (2 μg DNA)
miR-21	−0.23	−0.55
miR-222	−0.45	−0.46
miR-145	−0.17	−0.17

### Expression pattern of target mRNA *in vitro*due to transient transfection by HBx and in stable HBV producing cells

**Figure 2:**
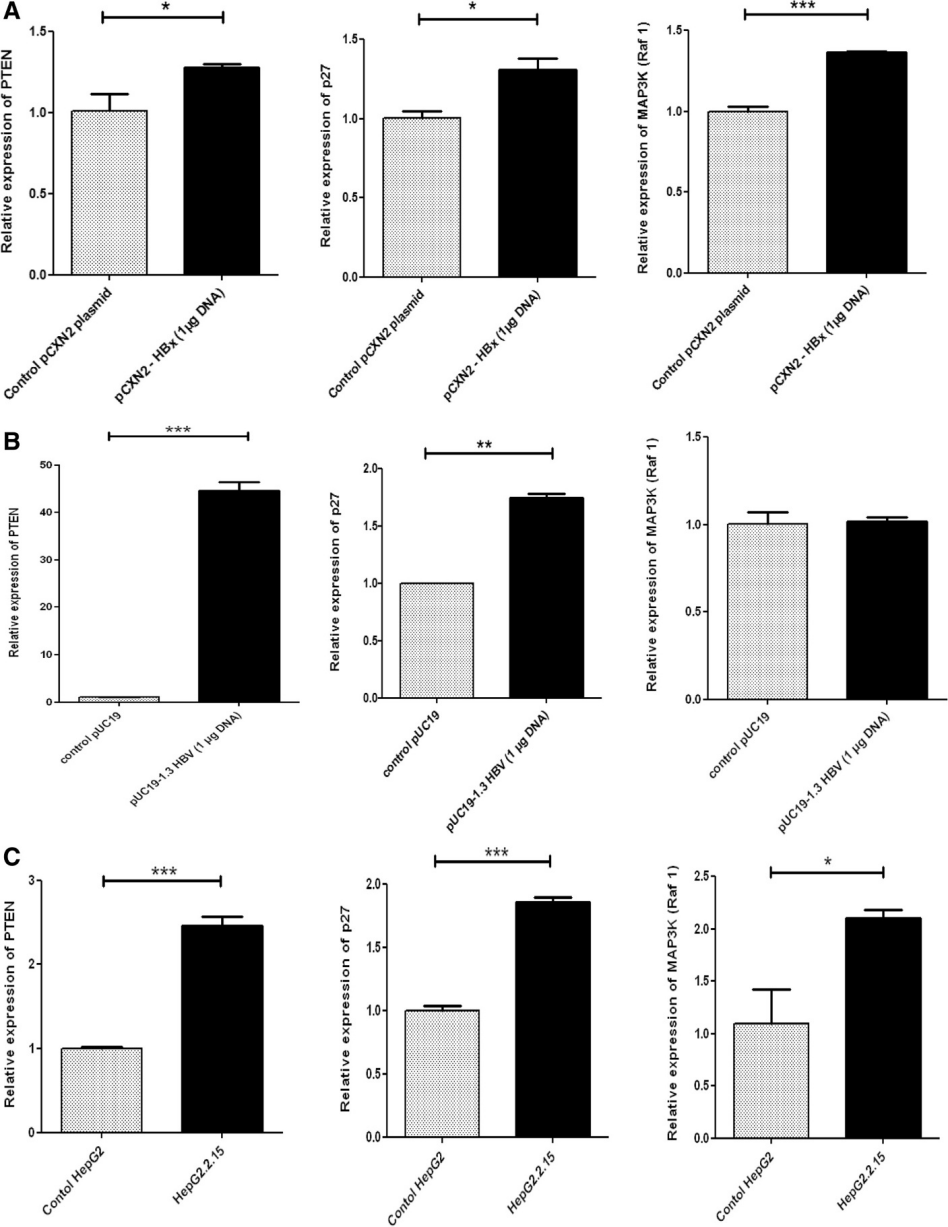
**Expressions of target mRNAs in HBx and HBV transfected and constitutively HBV synthesizing hepatoblastoma cells. (A)** Relative expressions of PTEN, p27 and MAP3K (Raf 1) - targets of miR-21, miR-222 and miR-145 respectively in HBx transfected HepG2 cells. Cells were transfected with 1 μg of pCXN2-HBx or pCXN2 as a control. **(B)** Relative expressions of PTEN, p27 and MAP3K (Raf 1) in 1.3 fold full length HBV genome transfected HepG2 cells. Cells were transfected with 1 μg pUC19-HBV or 1 μg pUC19 control vector. **(C)** Relative expressions of PTEN, p27 and MAP3K (Raf 1) in HepG2.2.15 cell line. RNA were extracted 48 hours post transfection. The mRNA expressions were measured by qRT-PCR and the expressions were normalized to GAPDH. Data are expressed as the mean ± SD from three independent experiments (*P < 0.05, **P < 0.01, ***P < 0.001; Student’s t-test).

Transfection of HepG2 cells with HBx caused up regulation of PTEN, p27 and MAP3K mRNA compared to control cells i.e. cells transfected with empty expression vector (Figure 2A). This result is consistent with experimental results observed with miRNA in HBx transfected HepG2 cells i.e. down regulation of miRNA in HBx transfected cells resulted in up regulation of target mRNAs. Transfection of HepG2 cells with 1.3 fold HBV genome produced the same result. All the target mRNAs- PTEN, p27 and MAP3K were found up regulated in 1.3 fold HBV genome transfected HepG2 cells when compared with HepG2 cells transfected with empty pUC19 vector (Figure 2B). However, the up regulation of MAP3K mRNA in HBV genome transfected HepG2 cell was marginal. When we measured the expression of PTEN, p27, and MAP3K mRNA in HepG2.2.15 cell line, we found that expression of all the target mRNA were elevated as compared to control HepG2 cells (Figure 2C). Expression of GAPDH was measured as internal control.

### Confirmation of expression of target gene at protein level by western blot due to transfection of HepG2 cells by HBx

**Figure 3:**
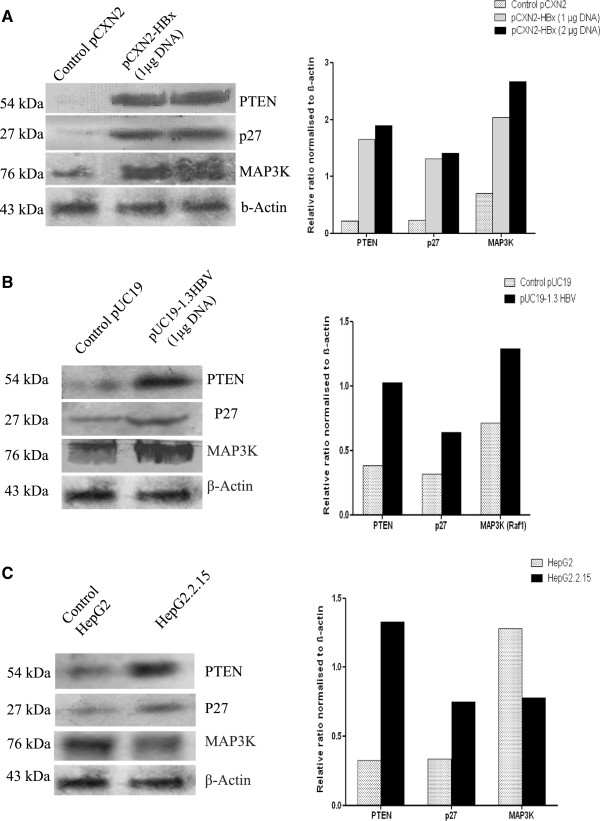
**HBx modulated expressions of target proteins in transiently transfected and HBV producing malignant hepatocytes. (A)** Western blot confirmed proteins PTEN, p27 and MAP3K (Raf 1) – targets of miR-21, miR-222 and miR-145 respectively were increased accordingly in HBx transfected HepG2 cells. Cells were transfected with 1 μg and 2 μg of HBx plasmid respectively or pCXN2 as a control plasmid. **(B)** Expressions of PTEN, p27 and MAP3K (Raf 1) proteins in 1.3 fold HBV transfected HepG2 cells. HepG2 cells were transfected with 1 μg pUC19-HBV or 1 μg pUC19 vector as a control. **(C)** Western blot exhibited differential expression of target proteins PTEN, p27 and MAP3K (Raf 1) in constitutively HBV producing HepG2.2.15 and control HepG2 cell line. Cells were collected for protein analysis 48 h after each transfection. β actin was taken as endogenous control. Protein bands were quantified using dentiometric scanner (Bio-Rad). Below are the graphical representation of intensity ratio between target proteins (PTEN, p27 and MAP3K (Raf 1)) and β actin in each lane.

Western blot analysis further confirmed that transfection by HBx caused up regulation of target protein compared to control cell line. These results were applicable for PTEN, p27 and MAP3K proteins - the targets of miR-21, miR-222 and miR-145 respectively (Figure 3A). Moreover, up regulation of protein expression in HepG2 cells caused by HBx was found to be dependent on the differential concentrations of HBx DNA. Higher the concentration of HBx plasmid during transfection, lower the expression of miRNA and in turn higher expression of proteins were found in HBx transfected cell line. When HepG2 cells were transfected with full length HBV genome, target protein expression were found to be higher in HBV transfected cells compared to empty vector transfected HepG2 cells (Figure 3B). Further, we compared target protein expression in HepG2.2.15 cell line and its control cell HepG2. We observed that PTEN and P27 were overexpressed in HepG2.2.15 cells whereas MAP3K (Raf1) expression was reduced (Figure 3C). Expression of β-actin was considered as endogenous control in these experiments.

### HBx differentially modulates onco miRNA (miR-21 and miR-222) and tumor suppressor miRNA (miR-145) expression as revealed by RNA interference experiments

**Figure 4:**
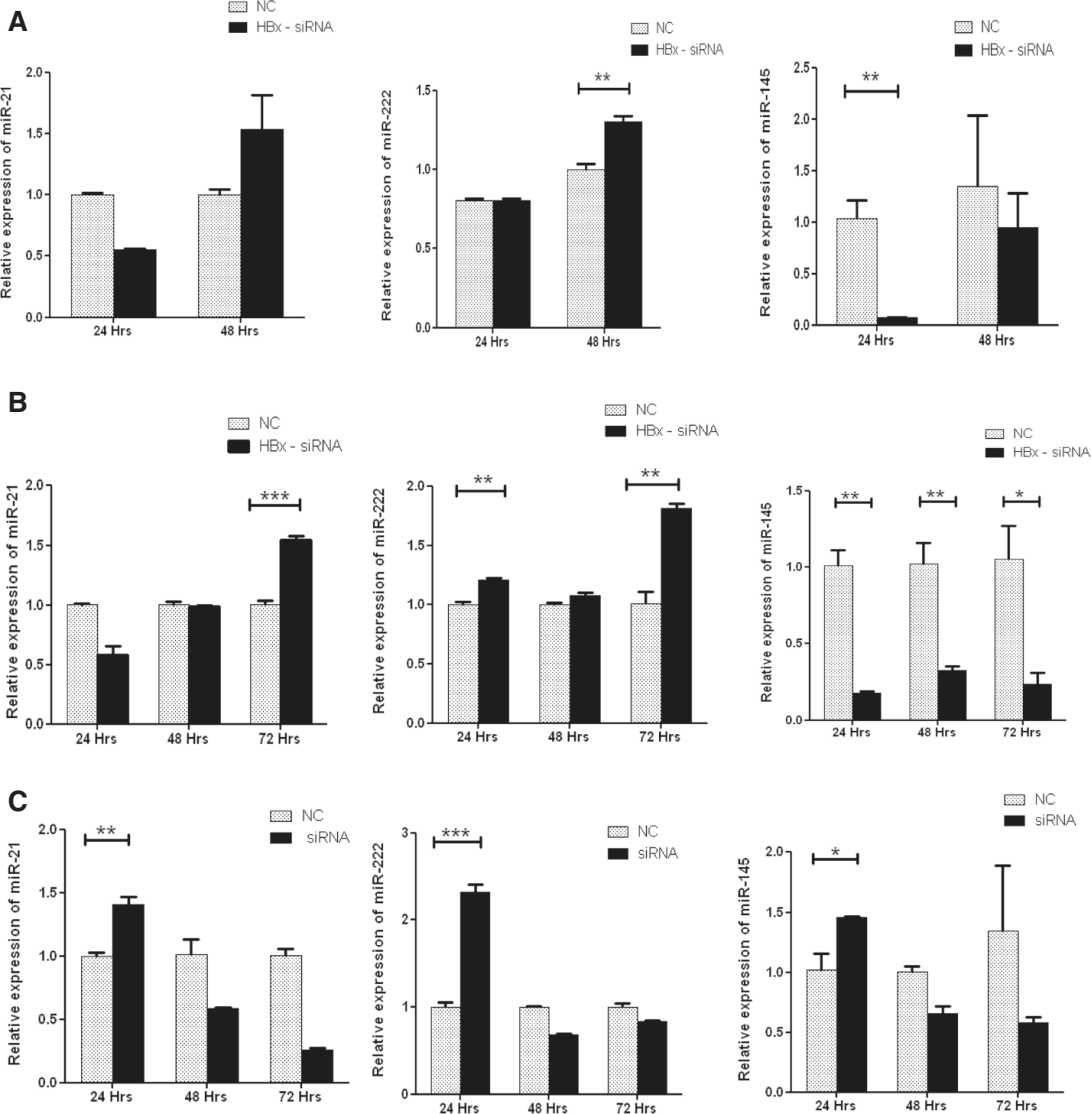
**miRNA expressions were restored after RNA interference study. (A)** Relative expressions of miR-21, miR-222 and miR-145 in HepG2 cells by co-transfection with HBx plasmid (0.8 μg) and HBx–siRNA (40 p mole) or negative control (NC). RNA were collected 24 and 48 h after each transfection. **(B)** Relative expressions of miR-21, miR-222 and miR-145 were measured when HepG2 cells were co-transfected with 1.3 fold full length HBV genome (0.8 μg) and HBx - siRNA (40 p mole) or negative control. RNA were collected 24, 48 and 72 h post transfection. **(C)** HepG2.2.15 cells were transfected with HBx siRNA (80 p mole) or negative control (NC) and relative expressions of miR-21, miR-222 and miR-145 were measured. RNA were collected 24, 48 and 72 h post transfection. Data plotted are the mean ± SD normalized to U6 expression. The experiments were performed in triplicate (*P < 0.05, **P < 0.01, ***P < 0.001; Student’s t-test).

HepG2 cells were co transfected with HBx plasmid; 1.3 fold full length HBV genome and HBV X gene specific siRNA to knock down the effect of HBx mRNA. HepG2.2.15 cells were also treated with HBx siRNA. In HBx transfected HepG2 cells, the expressions of two oncomiRNAs; miR-21 and miR-222 were found to be higher after 48 hours of transfection. The tumor suppressor miRNA miR-145 however, exhibited reduced expression in 24 and 48 hours (Figure 4A). In 1.3 fold HBV transfected HepG2 cells, miR-222 expression was restored 24, 48 and 72 hours post transfection whereas miR-21 expression was fully restored after 72 hours. miR-145 expression was not reestablished in HBV transfected HepG2 cells at any of the time points (Figure 4B). In HepG2.2.15 cell line, expressions of all the miRNAs were found reinstated 24 hour post transfection (Figure 4C).

### Expression levels of miRNAs among the HBV infected liver cirrhosis and hepatocellular carcinoma patients

**Figure 5:**
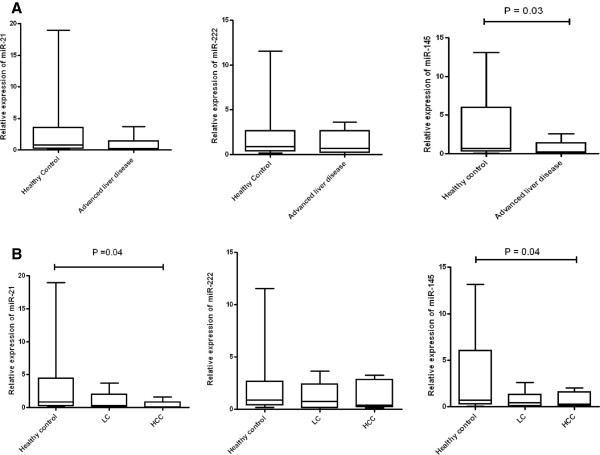
**miRNA expression in patient samples. (A)** Real-time PCR analysis of miR-21, miR-222 and miR-145 expression from patient serum samples. The miRNA levels in healthy controls were arbitrarily set as 1.0 and compared with advanced liver disease patients. **(B)** Comparison of miR-21, miR-222 and miR-145 expressions between healthy controls, LC and HCC patients. RNA was isolated from serum samples using miRVANA small RNA isolation kit and assayed using the TaqMan miRNA kit. The results were normalized to U6 endogenous control. Experiments were performed in triplicate. Error bars are means of ± standard deviation (SD). Mann–Whitney U test was performed to determine P-values (*P < 0.05, **P < 0.01, ***P < 0.001). LC = liver cirrhosis; HCC = Hepatocellular carcinoma.

The expressions of miR-21, miR-222 and miR-145 were decreased in advanced liver disease patients when these patients were compared with healthy controls (Figure 5A). These decreased expressions of miR-21, miR-222 and miR-145 were reflected in both LC and HCC patient groups when these two groups were compared separately with healthy controls (Figure 5B). Interestingly, the comparison indicated that the down regulation of miR-145 expression in advanced liver disease patients was significant (P = 0.0302). The down regulation of miR-21 and miR-145 expression in HCC patients was also found to be significant (P = 0.0487 in case of miR-21 and P = 0.0486 in case of miR-145) (Figure 5B).

## Discussion

Oncogenic roles of HBV X protein is well recognized as it affects the expression of cellular genes, which in turn alters the cell signaling and other cellular processes. HBx may render hepatocytes more vulnerable to other carcinogenic signals. These signals combined with host immune response and interaction of HBx with other cellular proteins, could substantially induce hepatocytic transformation [26–29] which advances towards HCC. Involvement of miRNAs is being uncovered in almost all aspects of cancer biology, such as proliferation, tumorigenesis, apoptosis, invasion/metastasis and angiogenesis [30, 31]. These small RNAs coordinate the interplay between complex signal transduction pathways. Several evidences depict that the expressions of miRNAs are remarkably modulated in malignant tissues due to multiple genomic and epigenetic alterations. Our present study provides evidence that HBx protein differentially modulates the expression of miRNA-21, miRNA-222 and miRNA-145 in hepatoblastoma cell lines. These findings were also validated using samples from LC and HCC patient.

It is now well acknowledged that a single miRNA can target more than one mRNA, likewise same mRNA can be a potential target of several miRNAs. In the present work, PTEN, p27 (Kip1) and MAP3K that have been suggested to be associated with cell proliferation, was studied as targets of miRNA-21, miRNA-222 and miRNA −145 respectively [14, 18, 19].

PTEN (phosphatase and tensin homolog), one of the targets of miRNA-21 acts as a phosphatase to dephosphorylate PIP_3_ resulting in the bisphosphate product PIP_2_. This dephosphorylation causes inhibition of the AKT signaling pathway. Our study revealed that miR-21, often described as onco miRNA, was found down regulated in HepG2 cells transfected with HBx plasmid. Similar results were also observed in other hepatoblastoma cell lines i.e., in HepG2.2.15 cell line and in 1.3 fold HBV transfected HepG2 cells. Use of HBx siRNA in 3 cellular system i.e. HBx transfected HepG2, full length HBV transfected HepG2 and HepG2.2.15 cell line demonstrated that miR-21 expression is recovered at various time points. Additionally, HBV related advanced liver disease patients which includes LC and HCC groups also exhibited down regulation of miR-21.

The p27 (Kip1) gene is a member of the Cip/Kip family of cyclin dependent kinase (CDK) inhibitors that binds to CDK2 and cyclin E complexes to prevent cell cycle progression from G1 to S phase [32]. Sage *et al.* [33] have demonstrated that miRNA – 221 & miRNA – 222 are responsible in cancer progression through the suppression of p27 (Kip1) expression. Our study depicted that miR-222 is down regulated in HBx and 1.3 fold HBV genome transfected HepG2 cells and in HepG2.2.15 cell line. Accordingly we found elevated level of p27 mRNA and protein expression in these transiently transfected HepG2 cell lines and in stably HBV reproducing cell line. Interestingly, miR −222 expression was restored in HBx transfected HepG2 cells (48 hours post transfection) and HBV transfected HepG2 cells (24, 48 and 72 hours post transfection) when they were treated with HBx specific siRNA. Similarly miR-222 expression was reinstated in X silenced HepG2.2.15 cells (24 hours post transfection). Our results indicate that HBx is instrumental in suppression of miR-222 expression, thereby increasing expression of p27. These events have been reported to cause cell cycle arrest in G1-S phase as evidenced by other group of researchers *in vitro* and in mouse primary hepatocytes [34, 35]. Furthermore, our study on HBV infected patients with different clinical outcomes (advanced liver disease patients or its subset LC and HCC patients) demonstrated that miR-222 expression was decreased, as compared to healthy controls.

Our study demonstrated that miR-145 was down regulated in HepG2 cells when transiently transfected with HBx plasmid and 1.3 fold HBV genome. Previous reports have suggested that HBx protein activates Ras–GTP complex and establishes Ras Raf MAP kinase signal cascade [36]. HBx was found to stimulate Ras-activating proteins of the Src family of tyrosine kinases also, which can signal to Ras [37]. Our result is in harmony with previous works as down regulation of miR-145 by HBx promoted up regulation of MAP3K (Raf 1) which plays an effective role in cell growth and proliferation by regulating downstream signaling cascade. Thus, our study showed that HBx promotes cell growth and proliferation by suppression of tumor suppressive miRNA-145.

However, in HepG2.2.15 cell line we observed miR-145 expression is higher compared to control HepG2 cells. We also found that though its target mRNA MAP3K (Raf 1) remained up regulated, MAP3K protein expression was reduced in HepG2.2.15 cells compared to its control cell line HepG2. A recent study by Jiang *et al.* [38] supports our results that miR-145 is up regulated by more than 5 fold in HepG2.2.15 than in HepG2 cells. In HepG2.2.15 cells, HBV DNA is carried as chromosomally integrated sequences and episomally as relaxed circular, covalently closed form of HBV genome [39]. There are evidences that chronic HBV induced HCC involves both HBV DNA integrated in host chromosome and covalently closed circular (CCC) episomal HBV genome. Genome integration into host chromosome exerts *cis* effects resulting in disruption and stimulation of cellular genes that are essential for cell growth and proliferation. On the other hand, *trans*-activating factors like HBx from episomal HBV DNA are responsible for cytoplasmic modulation of various signal transduction pathways leading to hepatocarcinogenesis [40]. We assume that the virus uses both the mechanisms in HBV induced HCC causing differential expression of miR-145 between transiently transfected HepG2 cells and HBV integrated HepG2.2.15 cell line. However, future study will clarify the exact mechanism involving modulation of miR-145.

One limitation of our study is that we could not compare liver tissue sample of HBV infected advanced liver disease patients with normal liver tissue. Most of the patients came with very critical condition of liver providing no option for biopsy. Furthermore, HepG2 is a hepatoblastoma cell line and differs from HCC cells on the basis of certain characteristics like histoclinical features, genetic alterations or activation of Wnt/β-catenin signaling. Though several *in vitro* studies have been accomplished with HepG2 cells, still use of hepatoma cells should be considered to investigate liver tumorigenesis.

To sum up, HBx differentially modulated expressions of miR-21, miR-222 and miR-145 in malignant hepatocytes. Reduced expression of these miRNAs was also observed in samples from advanced liver disease (LC and HCC) patients. Since our study was limited to the HBV genotype D, our results reflect the responses typical of this genotype. However, further studies are needed to verify the results in other genotypes.

## Conclusion

Current experimental evidence reveals that HBx protein differentially modulated the expression of miR-21, miR-222 and miR-145 and this modulation might be related to genotype D. Our findings provide new insight into possible way by which HBx protein acts through microRNA and thereby regulate host functioning. It will lead the way to targeted therapeutic new strategies for hepatic cancers. Interaction of cellular miRNA and HBx protein from other genotypes of HBV remains to be further investigated. Also the mechanistic approach will further clarify the reason behind the down regulation of these miRNAs caused by HBx protein.

## Abbreviations

AP: Activator protein
ATF: Activating transcription factor
CDK: Cyclin depandant kinase
CREB: cAMP response element-binding protein
DNA: De oxy ribo nucleic acid
HBV: Hepatitis B virus
HCC: Hepatocellular carcinoma
HBx: Hepatitis B virus X protein
JAK-STAT: Janus kinase-Signal transducer and activator of transcription
LC: Liver cirrhosis
MAP3K: Mitogen activated protein kinase kinase kinase
miRNA: MicroRNA(Ribo nucleic acid)
PBS: Phosphate buffer saline
PTEN: Phosphate and tensin homologue
PI3K: Phosphatidylinositide 3-kinase
RT-PCR: Real time polymerase chain reaction
TBP: TATA binding protein
TERT: Telomerase reverse transcriptase
TNF: Tumour necrosis factor
RNA: Ribo nucleic acid.
